# A survey of United States adult privacy perspectives and willingness to share real-world data

**DOI:** 10.1017/cts.2023.4

**Published:** 2023-02-01

**Authors:** Rachele M. Hendricks-Sturrup, Christine Y. Lu

**Affiliations:** 1 Department of Population Medicine, Harvard Pilgrim Health Care Institute and Harvard Medical School, Boston, MA, USA; 2 Duke-Robert J. Margolis, MD, Center for Health Policy, Washington, DC, USA; 3 Department of Interdisciplinary Health Studies, Ohio University, Athens, OH, USA

**Keywords:** Privacy, ResearchMatch, real-world data, real-world evidence, engagement

## Abstract

**Objective:**

Real-world data privacy is a complex yet underexplored topic. To date, few studies have reported adult perspectives around real-world data privacy and willingness to share real-world data with researchers.

**Methods:**

Relevant survey items were identified in the literature, adapted and pilot tested among a small convenience sample, and finalized for distribution. The survey was distributed electronically in April 2021 among adults (≥18 years of age) registered in ResearchMatch (www.researchmatch.org). Microsoft Excel was used to assess descriptive statistics across demographical items and four privacy-related items.

**Results:**

Of 402 completed responses received, half of respondents (∼50%) expressed willingness to share their prescription history data and music streaming data with researchers and unwillingness to share real-world data from several other sources. Most (53–93%) of participants expressed concern with five statements reflecting the sharing and use of their digital data online. Most participants (71–75%) agreed with four statements focused on individual measures taken to protect their personal privacy and disagreed (77–85%) with two statements centered on not being concerned about sharing or 3^rd^ party access to their personal data online.

**Conclusions:**

Our observations indicate an important yet unmet need to further explore and address real-world data privacy concerns among US adults engaging as prospective research participants.

## Introduction

In the 21^st^ century and perhaps beyond, the spectrum and concept of health data have and will continue to grow. This is equally true for the spectrum of ways in which health data can be generated, collected, stored, processed, and shared as evidence across entities and systems. The “patient-consumer spectrum” is a conceptual term that has emerged, being defined as the “phenomenon in which healthcare is rapidly transitioning from a periodic activity in fixed, traditional health care settings to an around-the-clock activity that involves the generation, use, and integration of data reflecting many aspects of individuals’ lives and behaviors” [1].

Like data along the patient-consumer spectrum, real-world data, often overlapping in definition with the term “big data,” are “data relating to patient health status and/or the delivery of health care routinely collected from a variety of sources” [2,3]. This comprises data from a wide variety of sources that include but are not limited to electronic health record (EHR) data, hospital or insurance company’s administrative and claims data, patient-generated data (e.g., data generated by in-home or self-monitoring devices such as wearables and fitness trackers), consumer-generated data, and laboratory data. Thus, given the largely unregulated nature of most real-world data sources, medical product regulators like the US Food and Drug Administration (FDA) have begun to suggest that clinical trial sponsors should consult with privacy experts to help them identify and address data privacy and security concerns when accessing data [4].

Health science and clinical research traditionally involves the collection of both survey and medical data. Yet, given that today billions of real-world data points can be generated from multiple and diverse sources by a single person, real-world data can be shared passively with researchers to understand clinical outcomes and health behavior in unprecedented ways, either before, following, or absent an intervention [5–7]. Moreover, sophisticated algorithms and data analytics procedures can be implemented to identify and recruit individuals who meet specific inclusion/exclusion criteria for a research study [8–10]. Analytical datasets comprised of real-world data are also augmenting existing clinical trial methodologies, such as through the use and development of external control arms for cancer and/or rare disease drug trials [11–13]. Despite these scientific advancements demonstrating the clinical and investigational utility of real-world data, the literature is scant in studies exploring adult perspectives around real-world data privacy in relation to their willingness to share their real-world data with researchers [14–17]. This study seeks to build on this nascent body of research.

## Methods

### Survey Development and Validation

A survey was developed and made available online using Qualtrics software. Parts of the survey contained four demographical items, three privacy threshold items, and two items focused on willingness to share data with researchers. Privacy-related survey items published by Seltzer *et al.* and validated and published by Doherty *et al.*, as well as demographic-related survey items validated and published by Zhu *et al.* were selected, adapted, and re-validated among a convenience sample of five individuals who identify as both patients and health consumers, for bias, relevance, and cognition [15–17]. Based on the pilot participants’ feedback, the survey questions were refined to improve item quality and clarity, and overall instrument clarity, appropriateness, and relevance.

A recent systematic review and a survey study each determined that age, income level, and education level are the strongest predictors of online or digital footprint activity [18,19]. Therefore, we applied Zhu *et al.*’s demographic data collection convention; demographic data collected included age, education level, duration of using online medical websites (years), and annual frequency of getting ill [16]. The final full survey consisted of 4 demographic items and 12 closed and 2 open-ended items focused on participants’ privacy concerns and perspectives (see full survey in Supplement).

### Survey Population

Adult survey participants were identified and contacted online using ResearchMatch, a “disease-neutral, Web-based recruitment registry to help match individuals who wish to participate in clinical research studies with researchers actively searching for volunteers throughout the US.” Populations in ResearchMatch live within the USA and Puerto Rico, are of all ages and races/ethnicities, and consist of healthy volunteers as well as those living with medical conditions. Individuals within the ResearchMatch database sign up to become volunteers through the ResearchMatch platform to support research studies.

### Survey Distribution

Access to the ResearchMatch platform for this study was provided through Ohio University. At the start of the study, a total of 148,090 participants were registered in ResearchMatch. In April 2021, the electronic survey was administered to adults (≥18 years of age) registered in the ResearchMatch database who agreed to be contacted to engage in the survey after receiving an informational electronic invitation letter via the ResearchMatch platform. As participants agreed to participate in the survey, they received an email correspondence with details about the study and link to the electronic survey.

A total of 13 searches were conducted in ResearchMatch to contact 19,499 ResearchMatch volunteers. Individuals were invited to participate in the survey, regardless of health status, race, gender, or any other mutable or immutable characteristics. Participants’ personal contact details were received only after participants agreed to participate in the survey. Participant email addresses were deleted or destroyed to prevent reidentification at the conclusion of the study.

### Survey Incentives and Completion Reminders

Financial incentives have been used in health research contexts to motivate participation and engagement (e.g., completing health risk assessments, health surveys, etc.) [20]. We offered individuals who completed the survey a random chance to receive a $250 (a total of two gift cards available), $100 (a total of four gift cards available), $50 (a total of six gift cards available), or $25 gift card (a total of 12 gift cards available). A random selection tool developed and deployed using Microsoft Excel to randomly select email addresses of survey participants and deliver the gift card incentives.

Survey participants were informed that their participation was entirely voluntary. No survey questions were mandatory, and participants were informed that they may skip any question(s) at any time. Survey participants were welcomed to contact the research team at any time with any questions or concerns about the study. Reminders were sent up to three times to participants who began but had yet to complete and submit the survey within the study timeframe.

### Data Analysis

The present analysis centers on a sample of ResearchMatch participants who completed the electronic survey. This analysis is part of a larger study examining US adult privacy-related experiences and motivations to share real-world data with researchers [21]. An online Qualtrics software tool [22] was used to calculate an ideal survey sample size (*n* = 384) based on the total ResearchMatch population (95% CI: 5% margin of error). Survey responses were assessed using descriptive statistics in Microsoft Excel.

### Ethics Review, Oversight, and Approval

ResearchMatch is a registry and collaborative project that is maintained at Vanderbilt University and overseen by the Vanderbilt University Institutional Review Board. The present study was reviewed and approved by the Ohio University Institutional Review Board under protocol #20-E-457. ResearchMatch participants’ completion of the survey implied their consent to engage in the survey.

## Results

### Overall Assessment

A total of 598 volunteers agreed to receive direct invitations to participate in the survey. Following receipt of email invitations, 470 participants initiated the survey and 402 completed and submitted the survey (86% completion rate among those who initiated the survey). Three participants who initiated but did not complete and submit the survey cited reasons for their non-completion, which were that the participant either did no't understand the way electronics affect health or did no't understand the nature of the survey questions. One participant noted that their age (>55) could be a factor as to why the participant did not understand the nature of the survey questions.

### Participant Characteristics

Table 1 summarizes demographic characteristics among all survey participants/respondents who completed or submitted the survey. Nearly all (99%) of the survey participants were over the age of 21. Most participants (87%) held either some college/associates/trade school, a bachelors’ degree, or masters’ degree. Just over half of participants (56%) reported to have used online medical websites for 7 years or more. Most participants (94%) reported an annual frequency of getting ill of six occurrences or less.

**Table 1.** Summary of survey respondents’ demographics (excluding non-responses)

	N (%) respondents
**Age range (years)**	
Under 20	3 (1)
21–30	63 (16)
31–40	78 (19)
41–50	57 (14)
51–60	71 (18)
Over 60	126 (31)
**Education level**	
High School	12 (3)
Some College/Associates/Trade School	98 (24)
Bachelors	138 (34)
Masters	117 (29)
Doctorate or other terminal degree	35 (9)
**Duration of using online medical websites (years)**	
Less than 1	18 (4)
2–3	36 (9)
4–5	53 (13)
6–7	38 (9)
More than 7	224 (56)
Never	5 (1)
Unsure	26 (6)
**Annual frequency of getting ill (number of occurrences)**	
Less than 1	193 (48)
2–3	149 (37)
4–6	35 (9)
7–10	5 (1)
More than 10	16 (4)

### Participant Willingness to Share Data with Researchers

Participants were asked to indicate their willingness to share diverse types of digital data with researchers. Table 2 presents a summary of participants’ responses, indicating that nearly half of respondents expressed willingness to share their prescription history data and music streaming data with researchers. For all other data types, less than half of the participants were willing to share such data with researchers. Most participants indicated unwillingness to share data from eight sources: email history, text message and phone call data, Google search history, online purchase history, tax records and income history, credit card statements, electronic medical records, and geolocation. Most participants did not use the following data sources: Twitter, Snapchat, and Yelp reviews and ratings.

**Table 2.** Summary of ResearchMatch participants’ willingness to share digital data with health researchers (excluding non-responses)

Data source	Yes - N (%)	No - N (%)	I do not use this source of data - N (%)
Facebook data	182 (45)	118 (29)	102 (25)
Twitter data	88 (22)	55 (14)	257 (64)
Instagram data	127 (32)	83 (21)	189 (47)
Snapchat data	48 (12)	72 (18)	278 (69)
Email history (Gmail, Yahoo, Comcast, Verizon, etc.)	131 (33)	264 (66)	5 (1)
Text message and phone call data	120 (30)	273 (68)	9 (2)
Google search history	171 (43)	228 (57)	2 (0)
Online purchase history (Amazon, Target, eBay, etc.)	185 (46)	211 (52)	5 (1)
Music streaming data (Spotify, Pandora, etc.)	195 (49)	104 (26)	103 (26)
Yelp reviews and ratings	126 (31)	72 (18)	201 (50)
Ride-sharing history (Uber, Lyft, etc.)	99 (25)	116 (29)	186 (46)
Fitness tracker data (Fitbit, Apple Watch, etc.)	181 (45)	67 (17)	154 (38)
Tax records and income history	78 (19)	309 (77)	14 (3)
Credit card statement data	76 (19)	302 (75)	23 (6)
Voting history	187 (47)	196 (49)	14 (3)
Prescription history (CVS, Walgreen’s, etc.)	202 (50)	189 (47)	9 (2)
Electronic medical record data	179 (45)	212 (53)	7 (2)
Geolocation (GPS from your phone or computer) data	155 (39)	226 (56)	19 (5)
Genetic data (23andMe, etc.)	136 (34)	141 (35)	124 (31)

### Online Data Sharing and Use

**Fig. 1.**
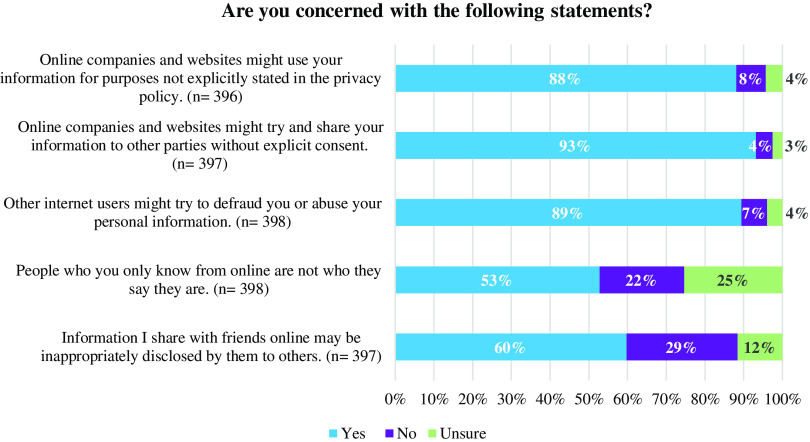
Participants’ concerns about online data use and sharing.

Participants were asked whether they were concerned with five statements reflecting the sharing and use of their digital data online (Fig. 1). The five statements centered on concern about 1) data use beyond what is stated within an online company’s or website’s privacy policy, 2) internet users defrauding or abusing personal information, 3) data sharing among companies and websites without expressed data subject consent, 4) inappropriate disclosure of data among online friends, and 5) false representation or identity among online users. Most (53–93%) of participants expressed concern with each of the five statements.

### Sharing Personal Information Online and Personal Privacy

**Fig. 2.**
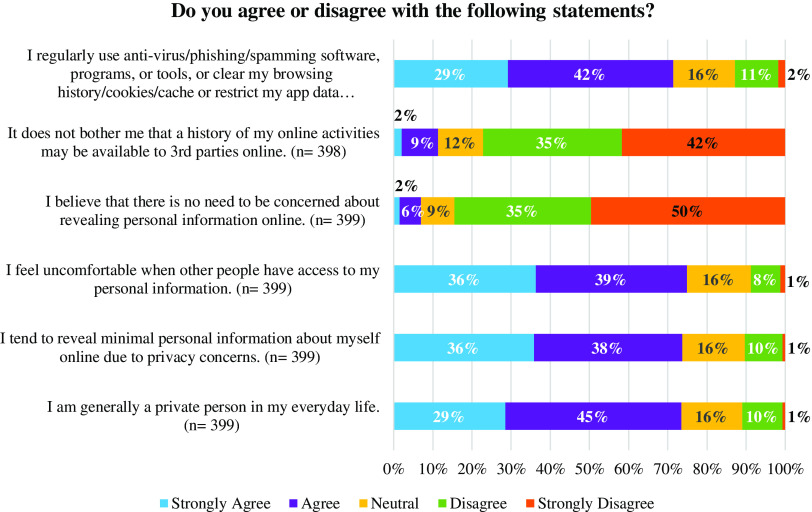
Participants’ concerns about revealing personal information online and personal privacy.

Participants were asked whether they agreed or disagreed with six statements focused on sharing personal information online (Fig. 2). Most participants (71–75%) either agreed or strongly agreed to 1) regularly using anti-virus/phishing/spamming software, programs, or tools, or exercising options to protect the privacy of their data (e.g., restricting app data permissions, etc.); 2) sharing minimal personal information about themselves online due to privacy concerns feeling uncomfortable when others have access to their personal information; 3) being a generally private person in everyday life, and 4) feeling uncomfortable when others have access to their personal information. Most participants (77–85%) either disagreed or strongly disagreed with the ideas that 1) it does not bother them that a history of their online activities may be available to 3^rd^ parties online and 2) there is no need to be concerned about sharing personal information online.

## Discussion

This study is one of the first studies to explore the real-world data privacy perspectives of a national sample of US adults. Many participants have taken at some point measures to protect their personal data privacy and expressed concern about sharing personal data or 3^rd^ party access to their personal data online. Participants were generally willing to share their prescription history data and music streaming data with researchers but were unwilling to share real-world data from several other sources. In practice, these negative experiences and limited data sharing preferences might function as a rate-limiting factor to successfully engaging prospective adult participants in potentially valuable, data-driven research. For instance, recent findings from our related study show that 1) privacy-related experiences may shape research participants’ willingness to share real-world data and 2) age range and education level may shape individuals’ willingness to share certain real-world data sources with researchers [21]. Yet, researchers may feel encouraged by further observations showing that negative privacy-related experiences are not associated with unwillingness to share most real-world data sources [21].

There are limitations to note for this study. Given the relatively low proportion of adults under age 20 engaged in this study, the findings may not be generalizable to adults in this general age demographic. Future work may explore the internal validity of our findings among this age group. Also, given that our study sample derived solely from ResearchMatch, a population that is likely or already somewhat engaged in data sharing for research, future work should explore whether our present findings might align with or differ from members of the general population who are not as engaged in research.

Our findings are partially consistent with observations in Seltzer *et al.*, who conducted very similar survey among their local patient cohort [15]. Interestingly, most participants in the present study and that of Seltzer *et al.* expressed willingness to share prescription history and music streaming data [15]. Although, a relatively greater proportion of participants expressed concerns about internet users defrauding or abusing their personal information and online companies and websites either sharing information with other parties without explicit consent and/or using information for purposes not explicitly stated in their privacy policies. Given that Seltzer *et al.*’s study was completed prior to the COVID-19 pandemic, the relatively greater proportion of participants’ privacy concerns might be related to the COVID-19 experience [15]. Recent studies show that individuals’ data privacy concerns were likely heightened during the COVID-19 pandemic, as privacy laws and regulations became relaxed to accommodate digital public health surveillance and remote health care [23,24].

Our findings likely indicate an unmet need to address participants’ privacy concerns about sharing their real-world data for research [14,15]. Immediate efforts to address these privacy concerns should include, as recommended during a recent National Academies of Science, Engineering, and Medicine expert convening [24]:

1. Ensuring privacy protections through comprehensive privacy laws and regulations.
2. Improving enforcements of third-party privacy policies and data use consent.
3. Facilitating education and engagement among industry and their third-party business associates, data subjects, and other key stakeholders in data governance and stewardship.
4. Implementing privacy-by-design or privacy enhancing technologies.
5. Curating and maintaining trustworthy data-sharing environments.
6. Addressing structural incentives that discourage electronic data sharing.

Like the FDA, industry and non-industry stakeholders, including but not limited to those engaged in the Personalized Medicine Coalition [25] and the Duke-Margolis Center for Health Policy’s Real-World Evidence Collaborative [26], have recently and continue to raise unresolved privacy concerns and other ethical considerations that follow or accompany acquisitions and uses of real-world data. Therefore, it would be both important and mission critical to engage research participants, including those engaged in ResearchMatch, in real-world evidence policy discussions, particularly in light of FDA’s guidance on use of real-world data in regulatory submissions for investigational new drug applications, and biologics license applications [27].

## Conclusion

US adults engaging as prospective participants in health research hold significant concerns about the privacy of their real-world data. Our study identified not only this but also an apparent need to balance such individuals’ real-world data privacy concerns with their impetus to engage in health research by sharing specific sources of their real-world data. Moving forward, opportunities to engage US adults as prospective health research participants in policy-related efforts intended to increase the acceptability of real-world evidence in regulatory submissions should not be missed.

## Supplementary material

For supplementary material accompanying this paper visit https://doi.org/10.1017/cts.2023.4. click here to view supplementary material
