# Supplementary material for: A survey of United States adult privacy perspectives and willingness to share real-world data
Source: J Clin Transl Sci. 2023 Feb 1;7(1):e64. doi: 10.1017/cts.2023.4 (PMC10052457; doi:10.1017/cts.2023.4)
Supplement: Supplementary file 1 [file S2059866123000043sup001.pdf]

INTRODUCTION

WELCOME!.
Thank you for participating in our study!

The purpose of this study is to determine how patients in ResearchMatch think and feel about many different types of electronic data, and the security and privacy of that data. We also want to learn how patients in ResearchMatch feel about donating different types of personal data to health research, now or in the future.

This survey contains 17 questions and should take around 10-15 minutes to complete. You can choose to not continue the survey at any time. You can also choose to not respond to or skip any question at any time.

Q1. This first question asks you to rate how various sources of digital data may be related to health. We describe this as “health-relatedness.” Please rate the health relatedness of sources of digital data on a scale of 0 to 10, with 0 being the least and 10 being the most.

	0 (Least)	1	2	3	4	5	6	7	8	9	10 (Most)
Electronic health record	○	○	○	○	○	○	○	○	○	○	○
Wearable fitness tracker	○	○	○	○	○	○	○	○	○	○	○
Commercial genetic profile	○	○	○	○	○	○	○	○	○	○	○

	0 (Least)	1	2	3	4	5	6	7	8	9	10 (Most)
Grocery store rewards card	○	○	○	○	○	○	○	○	○	○	○
Smartphone or another cellular device location	○	○	○	○	○	○	○	○	○	○	○
Photographs (from smartphone or another cellular device)	○	○	○	○	○	○	○	○	○	○	○
Internet browser history	○	○	○	○	○	○	○	○	○	○	○
Social media posts	○	○	○	○	○	○	○	○	○	○	○
GPS navigation (from car)	○	○	○	○	○	○	○	○	○	○	○
Emails	○	○	○	○	○	○	○	○	○	○	○
Online reviews	○	○	○	○	○	○	○	○	○	○	○
Social media activity	○	○	○	○	○	○	○	○	○	○	○
Text messages	○	○	○	○	○	○	○	○	○	○	○
Credit card statement	○	○	○	○	○	○	○	○	○	○	○
Home monitoring camera	○	○	○	○	○	○	○	○	○	○	○
Credit Report	○	○	○	○	○	○	○	○	○	○	○
Smart home thermostat	○	○	○	○	○	○	○	○	○	○	○
Voicemail	○	○	○	○	○	○	○	○	○	○	○
Call log	○	○	○	○	○	○	○	○	○	○	○
Electronic toll payment device	○	○	○	○	○	○	○	○	○	○	○

	0 (Least)	1	2	3	4	5	6	7	8	9	10 (Most)
Frequent flyer account	○	○	○	○	○	○	○	○	○	○	○

DSHAREWILLING

Q2.

Thank you for your responses. As a reminder, you can choose not to continue the survey at any time. Our next question asks about your willingness to share various types of data with researchers. Please note that we will not be collecting any of those types of data for this study.

Are you willing to share the following types of data with researchers today?

	Yes	No	I don't use this source of data
Facebook data	○	○	○
Twitter data	○	○	○
Instagram data	○	○	○
Snapchat data	○	○	○
Email History (Gmail, Yahoo, Comcast, Verizon, etc.)	○	○	○
Text Message and Phone Call Data	○	○	○
Google search history	○	○	○

	Yes	No	I don't use this source of data
Online purchase history (Amazon, Target, Ebay, etc.)	○	○	○
Music streaming data (Spotify, Pandora, etc.)	○	○	○
Yelp reviews and ratings	○	○	○
Ride-sharing history (Uber, Lyft, etc.)	○	○	○
Fitness tracker data (FitBit, Apple Watch, etc.)	○	○	○
Tax records and income history	○	○	○
Credit card statement data	○	○	○
Voting history	○	○	○
Prescription history (CVS, Walgreen's, etc.)	○	○	○
Electronic medical record data	○	○	○
Geolocation (GPS from your phone or computer) data	○	○	○
Genetic data (23andMe, etc.)	○	○	○

Data Health Relationship

Q3.

Thank you for your responses. As another reminder, you can choose not to continue the survey at any time. The following questions will ask you about the types of data as the question before, but instead of answering about willingness to share, we want to find out if you think each type of data is related to health.

For each data type, please select how much you agree or disagree that the data can be health related, rating from "Strongly Agree" to "Strongly Disagree."

For example, "Google search data" might be used to see if a patient with diabetes is searching for a new treatment or a blood glucose monitor. Another example might be if researchers collect social media posts that reflect one's health status or contain language that can reflect early signs of memory loss or other health conditions.

	Strongly Agree	Agree	Neutral	Disagree	Strongly disagree
Facebook data contain health-related information.	○	○	○	○	○
Twitter data contain health-related information.	○	○	○	○	○
Instagram data contain health-related information.	○	○	○	○	○
Snapchat data contain health-related information.	○	○	○	○	○

	Strongly Agree	Agree	Neutral	Disagree	Strongly disagree
Email accounts (Gmail, Yahoo, Comcast, Verizon, etc.) contain health-related information.	○	○	○	○	○
Text message data and phone call history contains health-related information.	○	○	○	○	○
Google search history contains health-related information.	○	○	○	○	○
Online purchase history (Amazon, Target, Google Buy, Ebay, etc.) contains health-related information.	○	○	○	○	○
Music streaming data (Spotify, Pandora, Apple Music, etc.) contains health-related information.	○	○	○	○	○
Electronic Medical Records contain health-related information.	○	○	○	○	○

	Strongly Agree	Agree	Neutral	Disagree	Strongly disagree
Yelp reviews and ratings contain health-related information.	○	○	○	○	○
Ride-sharing history (Uber, Lyft, etc.) contains health-related information.	○	○	○	○	○
Fitness Tracker/Wearables history contains health-related information.	○	○	○	○	○
Tax records and other income history contains health-related information.	○	○	○	○	○
Credit card statements contain health-related information.	○	○	○	○	○
Voting history contains health-related information.	○	○	○	○	○
Geolocation (GPS from your phone or computer) data contains health-related information.	○	○	○	○	○

	Strongly Agree	Agree	Neutral	Disagree	Strongly disagree
Genetic data (23andMe, etc.) contains health-related information.	○	○	○	○	○

Expectations

Q4.

Thank you for your responses. As a reminder, you can choose to not continue the survey at any time.
The following question will ask you about the type of feedback would you prefer to receive if you donated your electronic data to health researchers.

If you donated your electronic data to health researchers, what type of feedback would you prefer to receive? (Select all that apply)

- Feedback about the effects of my eating and exercise habits
- Feedback about how other habits contribute to my health
- Health-related language analysis of my social media posts
- Comparison my health data to other donors' data
- Potential risk factors for health conditions
- Other

Q4.1. If you selected "Other," then what types of feedback would you prefer to receive from researchers? If you did not select “Other,” then please type “NA.”

Knowing Risk

Q5.

Thank you for your responses. As a reminder, you can choose to not continue the survey at any time.

The following question will present scenarios to allow you to assess whether or not you would want to know the results of an analysis of your donated data.

For each of the following scenarios, please indicate whether or not you would want to know the results of an analysis of your donated data.

	I would want to know	I wouldn't want to know	Unsure
Patterns in your data indicate that you are at higher-than-average risk for a treatable disease, like high blood pressure or asthma.	○	○	○
Patterns in your data indicate that you are at higher-than-average risk for a non-treatable disease, like Alzheimers disease.	○	○	○

	I would want to know	I wouldn't want to know	Unsure
Patterns in your data indicate that you are at lower-than-average risk for any type of health condition.	○	○	○

Others Benefit

Q6.
Thank you for your responses. As a reminder, you can choose to not continue the survey at any time.
The following section contains questions asking you about how you feel you and/or others may be affected by you sharing your data.

Do you think others could benefit from you donating your electronic data?

Yes

Maybe

No

Data Concern

Q7. Do you have or have you had any concerns about donating your electronic data to researchers today or in the future? If yes, please describe your concerns here. If not, please state “no” and elaborate as to why.

Share Data

Q8. If you donated your electronic data to researchers, with whom would you want insights from your data to be shared with? (Select any that apply)

- Myself
- Family
- Researchers
- Doctor/healthcare provider
- Social network
- Others with health conditions like mine

Donate Upon Death

Q9. If the opportunity arose in the future, would you consider donating your electronic data to health researchers after death?

- Yes, all of my data
- Yes, but only some of my data
- No
- Unsure

Donate Upon Death - Data Type

Q10.

Which types of data would you feel comfortable donating after death? (Check all that apply)

- Facebook data
- Twitter data
- Instagram data
- Snapchat data
- Email History (Gmail, Yahoo, Comcast, Verizon, etc.)
- Text Message and Phone Call Data
- Google search history
- Online purchase history (Amazon, Target, Ebay, etc.)
- Music streaming data (Spotify, Pandora, etc.)
- Yelp reviews and ratings
- Ride-sharing history (Uber, Lyft, etc.)
- Fitness tracker data (FitBit, Apple Watch, etc.)
- Tax records and income history
- Credit card statement data
- Voting history
- Prescription history
- Electronic medical record data
- Geolocation (GPS from your phone or computer) data
- Genetic (23andMe, etc.) data

Privacy Threshold

Q11.

Thank you for your responses. As a reminder, you can choose to not continue the survey at any time.
This section will ask you questions about your opinions and beliefs about various types of online activities.

Are you concerned with the following statements?

	Yes	No	Unsure
Information I share with friends online may be inappropriately disclosed by them to others.	○	○	○
People who you only know from online are not who they say they are.	○	○	○
Other internet users might try to defraud you or abuse your personal information.	○	○	○
Online companies and websites might try and share your information to other parties without explicit consent.	○	○	○

	Yes	No	Unsure
Online companies and websites might use your information for purposes not explicitly stated in the privacy policy.	◯	◯	◯

Q12.

Do you agree or disagree with the following statements?

	Strongly Agree	Agree	Neutral	Disagree	Strongly Disagree
I am generally a private person in my everyday life.	◯	◯	◯	◯	◯
I tend to reveal minimal personal information about myself online due to privacy concerns.	◯	◯	◯	◯	◯
I feel uncomfortable when other people have access to my personal information.	◯	◯	◯	◯	◯
I believe that there is no need to be concerned about revealing personal information online.	◯	◯	◯	◯	◯
It does not bother me that a history of my online activities may be available to 3rd parties online.	◯	◯	◯	◯	◯

	Strongly Agree	Agree	Neutral	Disagree	Strongly Disagree
I regularly use anti-virus/phishing/spamming software, programs, or tools, or clear my browsing history/cookies/cache or restrict my app data permissions.	○	○	○	○	○

Q13.

Have any of the following events happened to you online?

	Yes	No	Unsure
An account of yours was accessed by someone you didn't give permission.	○	○	○
The privacy of your personal information was violated.	○	○	○
Your reputation was negatively affected as a result of information posted online.	○	○	○
You had an unpleasant experience as a result of information you gave out online.	○	○	○

	Yes	No	Unsure
You were the victim of fraud and/or identity theft.	○	○	○

Demographics

Q14.
Thank you for your responses. As a reminder, you can choose to not continue at any time.
This final section contains questions that ask you about your demographics.
Please make a selection from each category as it applies to you.

Age (years)

Under 20

21 to 30

31 to 40

41 to 50

51 to 60

over 60

Q15. Education Level

Middle school and below

High school

Some College/Associates/Trade School

Bachelors

Masters

Doctorate or other terminal degree

Q16. Duration of using online medical websites (years)

Less than 1

2 to 3

4 to 5

6 to 7

More than 7

Never

Unsure

Q17. Annual frequency of getting ill

Less than 1

2 to 3

4 to 6

7 to 10

More than 10

Powered by Qualtrics
